# Accuracy of Genomic Predictions for Resistance to Gastrointestinal Parasites in Australian Merino Sheep

**DOI:** 10.3390/genes16020159

**Published:** 2025-01-26

**Authors:** Brenda Vera, Elly A. Navajas, Elize Van Lier, Beatriz Carracelas, Pablo Peraza, Gabriel Ciappesoni

**Affiliations:** 1 Sistema Ganadero Extensivo, INIA Las Brujas, Ruta 48, km 10, Canelones 90200, Uruguay; bvera@inia.org.uy (B.V.); enavajas@inia.org.uy (E.A.N.); bcarracelas@inia.org.uy (B.C.); peraza@inia.org.uy (P.P.); 2 Departamento de Producción Animal y Pasturas, Facultad de Agronomía, Universidad de la República, Avda. Garzón 780, Montevideo 12900, Uruguay; evanlier@fagro.edu.uy; 3 Estación Experimental Facultad de Agronomía Salto, Ruta 31, km 21, Salto 50000, Uruguay

**Keywords:** *Ovis aries*, FEC, *Haemonchus contortus*

## Abstract

Infection by gastrointestinal nematodes (GINs) in sheep is a significant health issue that affects animal welfare and leads to economic losses in the production sector. Genetic selection for parasite resistance has shown promise in improving animal health and productivity. This study aimed to determine if incorporating genomic data into genetic prediction models currently used in Uruguay could improve the accuracy of breeding value estimations for GIN resistance in the Australian Merino breed. This study compared the accuracy of breeding value predictions using the BLUP (Best Linear Unbiased Prediction) and ssGBLUP (single-step genomic BLUP) models on partial and complete data sets, including 32,713 phenotyped and 3238 genotyped animals. The quality of predictions was evaluated using a linear regression method, focusing on 145 rams. The inclusion of genomic data increased the average individual accuracies by 4% for genotyped and phenotyped animals. For animals with genomic and non-phenotyped data, the accuracy improvement reached 8%. Of these, one group of animals that benefited from an ssGBLUP evaluation came from a facility with a strong connection to the informative nucleus and showed an average increase of 20% in their individual accuracy. Additionally, ssGBLUP slightly outperformed BLUP in terms of prediction quality. These findings demonstrate the potential of genomic information to improve the accuracy of breeding value predictions for parasite resistance in sheep. The integration of genomic data, particularly in non-phenotyped animals, offers a promising tool for enhancing genetic selection in Australian Merino sheep to improve resistance to gastrointestinal parasites.

## 1. Introduction

Parasitism by gastrointestinal nematodes (GINs) represents one of the main problems for sheep production, with *Haemonchus contortus* being the most prevalent parasite in Uruguay [1]. The selection of sheep resistant to GINs in Uruguay is possible using estimated breeding values (EBVs), based on the parasite egg count per gram of feces (FEC), which has allowed us to observe significant genetic gains [2]. The estimation of EBVs for FEC is based on genealogical and phenotypic information collected at the Núcleo Ultrafino de Glencoe (NUG) and at the Estación Experimental Facultad de Agronomía Salto (EEFAS), among other studs that participated in the Population Genetic Evaluation (PGE) of the Australian Merino breed raised in Uruguay. This name comes from the fact that, historically and to this day, the breed has received rams and semen from Australia [3]. However, one of the difficulties faced when running breeding programs is the need to have phenotype data from all animals, due to the associated costs involved and the difficulties in measuring traits such as FEC.

In contrast to conventional selection, which uses EBVs obtained from phenotypic and pedigree information using the best linear unbiased prediction method (BLUP) [4], genomic selection (GS), proposed by Meuwissen et al. [5], takes advantage of genomic data from single nucleotide polymorphisms (SNPs) to estimate genomic breeding values (GEBVs). Greater genetic gains have been reported with GS, increasing selection accuracy, especially in young animals and in difficult-to-measure traits with low heritability [6]. This methodology has already been implemented in several production systems in different farm animal species, including small ruminants [7,8]. In the case of sheep, GS is already being used in countries such as Australia [9], France [10], New Zealand [11], and the United Kingdom [12], to mention a few, focusing on the improvement of productive traits. Regarding genomic selection for resistance, both New Zealand and Australia have incorporated genomics into the selection of more resistant animals. Cunha et al. [13] report on these advances and other studies exploring the incorporation of genomics to improve prediction accuracy in breeding programs. However, in the Americas, its application in sheep breeding programs is still incipient, with Uruguay being one of the forerunners in the region in including GS into these programs.

Genomic selection for health traits such as GIN resistance could have a positive impact on animal welfare and increase economic benefits for producers. This is essential, given that parasitism not only causes economic losses, but also affects sheep health and welfare and leads to increased use of anthelmintics [14,15], which will increase selection for resistant parasites. Climate change is expected to exacerbate these issues by affecting sheeps’ ability to manage GIN infections, altering parasite life cycles, and increasing pasture contamination by GINs [16]. This underscores the need for alternative strategies, such as genetic selection for resistance to GINs, to be incorporated into sustainable parasite management programs.

The objective of this study was to assess the impact of including genomic information on the estimation and precision of breeding values in Australian Merino sheep in Uruguay. For this purpose, the data were analyzed in subsets together with the complete dataset, and EBVs and GEBVs were estimated using BLUP and single-step genomic best linear unbiased prediction (ssGBLUP) [17]. The impact of including genomic information in the GEBVs and individual accuracies was evaluated in groups of animals with and without phenotypes. In addition, the predictive abilities of the BLUP and ssGBLUP methods were compared using precision and bias statistics within a focal group of animals.

## 2. Materials and Methods

### 2.1. Phenotypic and Pedigree Data

A total of 32,713 Australian Merino animals, born between 2001 and 2022, had FEC phenotypes recorded across 13 studs that participated in Uruguay’s genetic evaluation (https://www.geneticaovina.com.uy/, accessed on 14 December 2024). FEC sampling was performed according to the protocol used for genetic evaluations [2], but only the first record was included in our study. Further details of the sampling protocol are described in Vera et al. [18] and Ciappesoni et al. [19] Briefly, lambs were treated with an anthelmintic at weaning and then FEC was monitored until an average FEC of 500 eggs per gram of feces was reached, with no more than 20% of individuals with zero counts. At that point, all lambs were sampled, with fecal samples taken directly from the rectum and sent to the parasitology laboratories, where FEC were measured using a modified McMaster technique with a sensitivity of 100 eggs per gram of feces [20]. Due to the non-normal distribution of FEC, the natural logarithm of FEC was used as the analyzed variable, calculated as LogFEC = Log_e_(FEC + 100) [2]; for simplicity, LnFEC. Descriptive statistics are presented in Table 1.

Pedigree data of 32,713 animals were obtained from the sheep national database of the Secretariado Uruguayo de la Lana, which comprised information provided by the Uruguayan Rural Association and the Uruguayan Australian Merino Breeders Association.

### 2.2. Genotypic Data

A total of 3238 animals were genotyped using at least one medium-density panel: GeneSeek^®^ Genomic Profiler™ BeadChip (GGP, GeneSeek, Lincoln, NE, USA), Illumina OvineSNP50 BeadChip, Illumina 15K (Illumina, San Diego, CA, USA), and Axiom^®^ Ovine 60K (Thermo Fisher Scientific, Waltham, MA, USA). Genotypic data were imputed to the GGP array using FImpute v3.0 software [21], since the highest number of animals was genotyped with this panel. Genotyped animals represent 10% of the total population (Table 2).

Quality control of the genotypic data was performed using the preGSf90 of the BLUPF90 family programs [22]. Single nucleotide polymorphisms (SNPs) with a call rate below 0.90, and SNPs with a minor allele frequency (MAF) lower than 0.05 were excluded. Animals with a call rate lower than 0.90 were also removed from the analysis. Pedigree registration errors were corrected based on genomic information using Seekparentf90 program to detect incompatibilities between parents and offspring based on mendelian conflicts counts [23]. After quality control, 3143 individuals and 37,741 SNPs were used in this study.

### 2.3. Animal Model and Genetic Parameters Estimation

The following univariate animal model was used to estimate (G)EBVs and variance components for the calculation of heritability:

y=Xb+Za+e

where y, b, a, and e are vectors for FEC phenotypes, fixed effects, additive genetic effects, and random errors, respectively. X and Z are incidence matrices for b and a, respectively. Fixed effects include 494 contemporary groups (birth year, gender, farm, and management flock), type of birth (two levels: single and multiple), dam age (three levels: 2, 3, and ≥4 years old), and the age of the animal at FEC recording as covariate.

For variance components estimation with BLUP and ssGBLUP, the average information restricted maximum likelihood algorithm (AIREML) was used, described in Jensen et al. [24] and included in BLUPF90 family of programs (AIREMLF90) [22]. Heritability ($h^{2}$) for the FEC trait (LnFEC) was estimated as $h^{2}=\frac{\sigma_{a}^{2}}{\sigma_{y}^{2}}=\frac{\sigma_{a}^{2}}{\sigma_{a}^{2}+\sigma_{e}^{2}}$, where $\sigma_{a}^{2}$ corresponds to additive genetic variance, $\sigma_{y}^{2}$ to phenotypic variance, and $\sigma_{e}^{2}$ to residual variance.

### 2.4. EBVs and GEBVs Prediction

The EBVs were estimated using pedigree information based on the BLUP method [25], where $a∼N\left(0,A\sigma_{a}^{2}\right)$, with A being the additive relationship matrix from pedigree records and $\sigma_{a}^{2}$ being the additive genetic variance.

The ssGBUP methodology was used for the estimation of GEBVs. In this method, the A and G matrices are combined to generate the H matrix. The inverse of the matrix ($H^{-1}$), refs. [17,26] is defined as follows:

$${H^{-1}=A}^{-1}+\left(\begin{array}{cc} 0 & 0\\ 0 & G^{-1}-A_{22}^{-1} \end{array}\right)$$

where A^−1^ is the inverse of the pedigree-based relationship matrix, and G^−1^ is the inverse of the genomic relationship matrix that was constructed as in VanRaden [27] using observed allele frequencies; ($A_{22}^{-1}$) is the inverse of the pedigree-based relationship matrix for genotyped animals. To evaluate the compatibility between the G and A_22_ matrices, the off-diagonal elements of the numerator relationship matrix for genotyped animals ($A_{22}^{-1}$) and the weighted genomic relationship matrix (G*) obtained by preGSf90 were compared. The Pearson correlation coefficient between the two vectors was calculated.

### 2.5. Individual Accuracies

EBVs’ accuracies were estimated for each individual as a function of the prediction error variance (PEV) and incorporating the inbreeding coefficient *F* as described in Aguilar et al. (2020) [28]:

$$r=\sqrt{1-\frac{PEV}{\left(1+F_{i}\right)\sigma_{a}^{2}}}$$

where PEV is the prediction error variance; Fi is a measure of inbreeding from A or from H; and $\sigma_{a}^{2}$ is the additive genetic variance of the trait.

### 2.6. Predictability

1. Forward prediction

**Figure 1:**
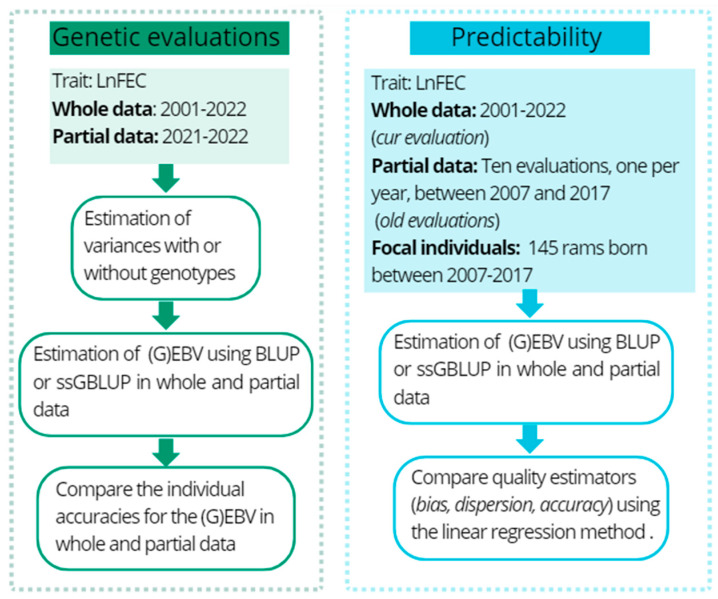
Workflow for comparing individual accuracies and quality estimators between BLUP and ssGBLUP models.

The genomic predictions were validated by forward predictions, where the prediction for young animals using truncated or partial data was compared to GEBVs with whole data (Table 2). For this, the 2021–2022 generations had their phenotypes masked (FEC = 0). (G)EBVs and individual accuracies were estimated on whole (2001–2022 generations) and partial (2021–2022 generation) data using BLUP and ssGBLUP models (Figure 1).

Pearson correlations were performed for breeding values of animals born in 2021–2022 estimated without phenotypic data (sub-index *p*) and those obtained in a whole evaluation (sub-index *w*) using BLUP and ssGBLUP.

2. Linear regression method

In this study, we were interested in evaluating bias, dispersion, and accuracy of rams at the time of their selection, i.e., at birth before they have progeny with records. For this, 145 rams were selected as a focal group and the LR method was used [29] on the two previously described datasets, whole data (subscript *w*) and partial data (subscript *p*). The partial dataset (old evaluation) can be interpreted as the evaluation at the time of selection decisions, and the whole dataset (current evaluation) as a posteriori confirmation of the goodness of these selection decisions [30].

For this purpose, EBVs from a genome-based method (ssGBLUP) and a pedigree-based method (BLUP) were calculated for 145 rams born between 2007 and 2017 from two scenarios: (1) Partial dataset, animals with individual phenotype and without progeny information (e.g., when they were lambs); (2) whole dataset, evaluation with all the individuals including progeny information and phenotypes (Figure 1).

In this paper, we will use the symbols ${\hat{u}}_{p}$ or EBV_p_ to refer to the EBV estimated with less information (or partial dataset) and ${\hat{u}}_{w}$ or EBV_w_ to refer to the EBV estimated with more information (or whole dataset). The LR method estimators used in this study are shown below (refer to Legarra and Reverter, 2018 [29] for a detailed description of all estimators).

$$\mathrm{Bias}\;({\hat{∆}}_{p})$$

Bias is obtained as the difference between the mean EBVp and the mean EBVw, with a zero expected value if evaluation is unbiased: ${\hat{∆}}_{p}={\hat{u}}_{p}-{\hat{u}}_{w}$

$$\mathrm{Dispersion}\;({\hat{b}}_{p})$$

This statistic is the estimator of EBVs dispersion and corresponds to the slope of the regression of EBVw (computed with the whole dataset) on estimated EBVp (computed with the partial dataset), with an expected value of one. Values of ${\hat{b}}_{p}$ < 1 indicate over-dispersion and ${\hat{b}}_{p}$ > 1, under-dispersion. ${\hat{b}}_{p}=\frac{cov\left({\hat{u}}_{p},{\hat{u}}_{w}\right)}{var\left({\hat{u}}_{p}\right)}$

$$\mathrm{Ratio}\;\mathrm{of}\;\mathrm{accuracies}\;({\hat{p}}_{w,p})$$

This estimator calculates the relative increase in accuracy from EBVp to EBVw. A higher value indicates a small improvement in accuracy, while a lower value suggests a higher increase in accuracy when including phenotypic data in genetic evaluations.

$${\hat{p}}_{w,p}=\frac{cov\left({\hat{u}}_{p},{\hat{u}}_{w}\right)}{\sqrt{var\left({\hat{u}}_{p}\right)var\left({\hat{u}}_{w}\right)}}$$

$$\mathrm{Ratio}\;\mathrm{of}\;\mathrm{reliabilities}\;({\hat{p}}_{w,p}^{2})$$

This estimator represents the inverse of the reliability increments from EBVp to EBVw and is obtained as the slope of the regression of EBVp on EBVw.

$${\hat{p}}_{w,p}^{2}=\frac{cov\left({\hat{u}}_{p},{\hat{u}}_{w}\right)}{var\left({\hat{u}}_{w}\right)}$$

## 3. Results and Discussion

### 3.1. Variance Components and Genetic Parameters for FEC

The heritability estimates for FEC obtained using the BLUP and ssGBLUP methods were similar and relatively low (0.19 ± 0.01). Including genomic data in the estimation resulted in only a marginal increase, as detailed in Table 3.

These estimates are consistent with those reported by other researchers, which range from 0.1 to 0.3 [31,32,33,34,35]. The direct heritability estimate for FEC is lower than that reported by Safari et al. [36] but slightly higher than the estimate of 0.18 reported by Ciappesoni et al. [32]. Currently, this heritability estimate is used in national genetic evaluations for this breed and indicates a moderate improvement in the trait through selection. Unlike Ciappesoni et al. [32], this study is based on a larger number of phenotypic records (+19,379) and includes genomic information in the estimation of genetic parameters for FEC. These results confirm the parameters estimated by Vera et al. [18], with an increase in phenotypic (+6075) and genomic (+1538) data in this study.

### 3.2. Individual Accuracy in the Validation Population

The mean theoretical accuracies of the validation population (generation 2021–2022) were higher in ssGBLUP compared to BLUP. The higher theoretical accuracy of ssGBLUP is attributed to its lower prediction error.

**Figure 2:**
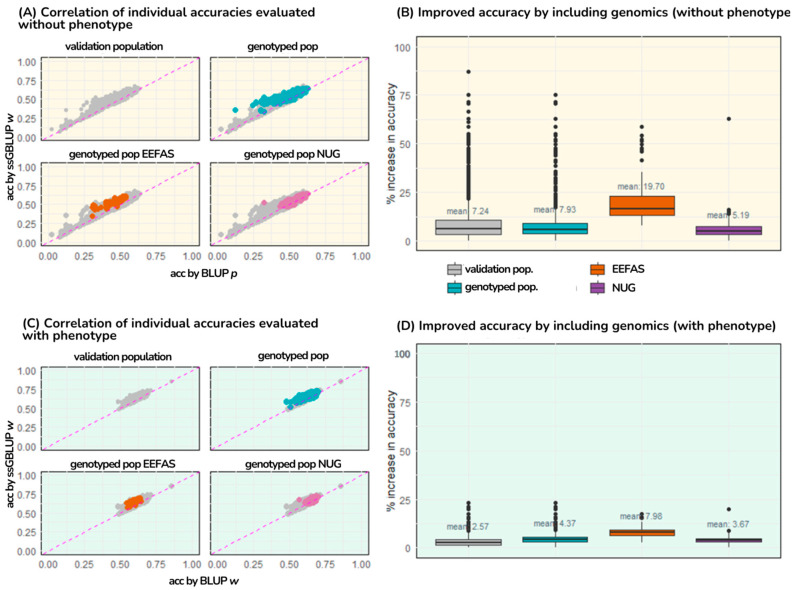
(G)EBV accuracies in validation population (**A**) without and (**C**) with FEC phenotype. Boxplot diagrams for accuracy increase using ssGBLUP compared to BLUP in evaluations (**B**) without phenotype and (**D**) with phenotype.

As shown in Table 4 and Figure 2, the accuracy of the validation population increased by 3% and 7% when genomic information was included in scenarios with and without phenotypes, respectively. Increases in accuracy using the ssGBLUP model compared to the BLUP model in dairy sheep populations have been reported [10], with 0.10–0.20 increases in traits other than FEC but with low to medium heritabilities.

In the validation population, 1059 genotyped animals exhibited average accuracy increases of 4% and 8% when molecular information was included in evaluations with and without phenotypes, respectively. Among these, animals genotyped from the informative nucleus (NUG) showed average accuracy gains of 3% and 5% when genomic information was included in phenotyped and non-phenotyped evaluations.

For the highly connected flock (EEFAS), evaluations that included phenotypic data resulted in an average increase of 8%, while those without phenotypic data exhibited an average increase of 20%. Notably, some animals in these evaluations experienced accuracy improvements of up to 60%.

It is known that accuracy increases when comparing genomic models with pedigree models are due to the correct estimation of genetic relationships between animals and to the increase in the partition of variance [37]. In this sense, increments in the validation population mean accuracy could be due to a larger additive relationship. Other studies have already shown that the ssGBLUP method increased prediction accuracies compared to the BLUP method [38,39,40]. EBVs were less accurate compared to GEBVs because they do not include information about the mendelian segregation residue. The genotyped population represents only 7% of total population and is not homogenously distributed, as it is concentrated in the last years (2015–2019).

Figure 2A shows that with BLUP without phenotypic data, accuracies range between 0 and 0.6, and when phenotype is included, accuracies are higher and range between 0.5 and 0.9 (Figure 2C). On the other hand, in ssGBLUP evaluations with and without phenotypic data, genotyped animals separate from the line are observed, in other words, their accuracy values increase when molecular information is included.

In summary, (G)EBV estimates are more accurate when based on multiple sources of information. In this study, genotyped animals showed greater increases in accuracy, with genotyped individuals from EEFAS exhibiting the highest average increases in accuracy compared to the rest of the animals. These results are relevant in PGE since unphenotyped animals from farms connected to informative nuclei could benefit from more accurate predicted GEBVs, resulting in a better selection of animals.

### 3.3. Quality of Genomic Predictions Using LR Method

ssGBLUP predictability was better compared to BLUP, with better quality estimators for the ssGBLUP model (Table 5). Both genetic and genomic evaluations presented in this study exhibited minimal bias, with BLUP (${\hat{∆}}_{p}$ = 0.0612) presenting slightly more. Bias is usually ignored in evaluations, but it has a great impact in practice [41,42]. Our results suggest that when evaluated with a BLUP model, all young animals (selection candidates) would show overestimates of 0.0612 and 0.0142 units in (G)EBVs when estimated with BLUP and ssGBLUP, respectively. More recent studies in dairy sheep in France show that by using metafounders to model missing pedigree, bias is low in genetic and genomic evaluations [43]. Other studies have reported that the ssGBLUP methodology including inbreeding is unbiased, but shows over-dispersion, possibly due to the data structure and the genotyped animals subset [40].

These estimators (accuracy, bias, and dispersion) improved slightly using the ssGBLUP model, which agrees with Macedo et al. [44], who concluded that the inclusion of molecular information in sheep evaluations increases EBVs accuracies in young rams. On the contrary, it has been reported that in the Latxa breed dairy sheep populations, no increases or decreases were observed in accuracies when including molecular information, but predictions were almost unbiased when using ssGBLUP [45].

In summary, both individual theoretical predictions and the quality of estimators were superior in ssGBLUP models. Furthermore, our results show that it is possible to predict the breeding value of non-phenotyped animals with acceptable accuracies using ssGBLUP models. For the population evaluated in this study, it would be interesting to consider other strategies to improve prediction quality, as studies on other species suggest that prediction accuracies can be significantly improved by using multi-trait models, particularly when a low heritability trait is correlated with a high heritability trait [46,47,48]. Another strategy is the use of metafounders (MFs) to model missing pedigree in genealogies, as it gives unbiased estimations in dairy sheep [49]. In addition, increasing the number of records for GIN resistance as well as the number of genotyped animals in the reference population would result in more accurate GEBV estimations due to higher and more trustful additive genetic (co)variances.

## 4. Conclusions

This study demonstrates that it is possible to predict the breeding values of non-phenotyped animals with acceptable accuracy using ssGBLUP models. In addition, the inclusion of genomic information may improve the accuracy of estimated breeding values for resistance to gastrointestinal nematodes.

Considering that the fecal egg count recording is time-consuming, costly, and often unattractive to breeders, the integration of molecular data into traditional evaluations, along with the prediction of estimated breeding values (EBVs) without phenotypic data, offers a promising alternative. This approach could be particularly beneficial for breeding programs that include the FEC trait, provided that a growing reference population is maintained.

## Figures and Tables

**Table 1:** Descriptive statistics of fecal egg counts (LnFECs) and age of animals at recording (days) from the total database (*n* = 32,713).

Trait	Mean	Standard Deviation	Minimum	Maximum
LnFEC	6.69	1.15	4.61	10.5
Age at FEC recording (days)	280.6	72.7	104	514

**Table 2:** Number of genotyped and non-genotyped animals with FEC phenotype in the training and validation population.

Group	Whole Data	Validation Population
Generation	2001–2022	2021–2022
Number of animals with records	32,713	5307
Number of animals with genotypes	3143	1059
Total number of animals	32,713	5307

**Table 3:** Estimated genetic parameters and heritability for FEC (LnFEC1) in Australian Merino sheep.

Methodology	$\sigma_{a}^{2}$	$\sigma_{e}^{2}$	$h^{2}$
BLUP	0.1591 ± 0.011	0.6742 ± 0.009	0.1909 ± 0.012
ssGBLUP	0.1606 ± 0.009	0.6724 ± 0.011	0.1928 ± 0.012

$\sigma_{a}^{2}$ = additive genetic variance, $\sigma_{e}^{2}$ = residual variance, $h^{2}$ = direct heritability.

**Table 4:** Mean accuracies for each subgroup in validation population.

		Unphenotyped			Phenotyped		
	N	EBV Acc	GEBV Acc	Δacc (%)	EBV Acc	GEBV Acc	Δacc (%)
Validation population	5307	0.423	0.452	7.2	0.613	0.629	2.5
Genotyped	1059	0.508	0.544	7.9	0.651	0.679	4.3
Genotyped EEFAS	136	0.442	0.525	19.7	0.610	0.659	7.9
Genotyped NUG	879	0.523	0.549	5.2	0.660	0.684	3.6

Δacc (%) = mean accuracy increase from EBV to GEBV.

**Table 5:** Quality estimators of prediction models.

Statistics	Statistics	Estimator	BLUP	ssGBLUP
Bias	${\hat{∆}}_{p}$	${\hat{∆}}_{p}={\hat{u}}_{p}-{\hat{u}}_{w}$	0.0612	0.0142
Dispersion	${\hat{b}}_{p}$	${\hat{b}}_{p}=\frac{cov({\hat{u}}_{p},{\hat{u}}_{w})}{var({\hat{u}}_{p})}$	0.7670	0.7865
Ratio of accuracies	${\hat{p}}_{w,p}$	${\hat{p}}_{w,p}=\frac{cov({\hat{u}}_{p},{\hat{u}}_{w})}{\sqrt{var({\hat{u}}_{p})var({\hat{u}}_{w})}}$	0.6136	0.6831
Ratio of reliabilities	${\hat{p}}_{w,p}^{2}$	${\hat{p}}_{w,p}^{2}=\frac{cov({\hat{u}}_{p},{\hat{u}}_{w})}{var({\hat{u}}_{w})}$	0.4909	0.5932

## Data Availability

Restrictions apply to the availability of these data. Data were obtained by INIA and are available from the authors with INIA’s permission.
